# The KT Jeang Retrovirology Prize 2020: call for nominations

**DOI:** 10.1186/s12977-019-0509-7

**Published:** 2020-02-10

**Authors:** Susan R. Ross, Johnson Mak, Ariberto Fassati

**Affiliations:** 1 Department of Microbiology and Immunology, University of Illinois College of Medicine at Chicago, 835 S Wolcot Rm E703 MC790, Chicago, IL 60612-7342 USA; 2 Institute of Glycomics, Griffith University, Gold Coast Campus, Parklands Drive, Southport, QLD 4222 Australia; 3 Division of Infection & Immunity, The Rayne Institute, University College London, 5 University Street, London, WC1E 6BT UK

The Retrovirology prize was initiated by our founding Editor Kuan-Teh Jeang (Teh). In his 2005 editorial (Retrovirology 2005 2:26), Teh argued that there were few prizes dedicated to the “silent majority” of mid-career scientists whereas prizes for younger or older colleagues were numerous. He concluded that a prize honouring the achievements of colleagues in the 45–60 age bracket in the field of Retrovirology would fill an important niche, and so the prize was started with a generous philanthropic donation from Kuan-Chen Jeang (who is also the sibling of Teh). After the untimely passing of Teh, the prize was renamed the KT Jeang Retrovirology prize to honour Teh’s contribution to our field. A total of 13 outstanding scientists have been awarded with Retrovirology Prize since its inception, and the full list is available here (https://www.biomedcentral.com/collections/retrovirology-prize). The achievements of our awardees have been described in the editorials that accompany the official announcement of the winner in the journal.

During the early years of Retrovirology Prize, nominations were initiated by our Associate Editors, which was subsequently extended to members of our editorial board to better reflect the wider community of our discipline. The voting process was carried out by secret ballot cast by the Associate Editors. Despite our good intentions, we now realize that there are areas of improvement that can be made to this process, to better reflect the diversity of our research community.

Therefore, in consultation with our Associate Editors, Susan Ross, Johnson Mak and I have established a new Retrovirology Prize committee of 12 members, consisting of 6 females and 6 males based in different regions of the world, to provide a fantastic pool of expertise (see below). Members of this committee include past awardees and individuals who are outside of the Retrovirology Editorial team. Appointment to this committee will be for 3 years and committee members cannot be nominated for the award during their tenure as assessors of the Retrovirology Prize. To maintain the original spirit of Retrovirology Prize, all applications will be assessed and scored based on the merit of the applicants’ demonstrated scientific accomplishments. We have also changed the nomination process to be more inclusive; nominations, including self-nominations, are open to anyone, please follow this link for instructions on how to nominate https://retrovirology.biomedcentral.com/about/retrovirology-prize.

One may ask why do we need a Retrovirology prize. One reason is to celebrate our champions and help them become role models for the younger generations. Another reason is to remind ourselves of key discoveries in the field and who made them. We all “stand on the shoulder of giants” but we all too often forget, in our preoccupation to see far into the distance, to take a look at those “giants” themselves. Yet another reason is to stir the community. In considering our colleagues’ work, who might deserve the prize and why, we benefit our community by forcing all of us to take stock and think, discuss and persuade others. So please consider nominating a deserving colleague for the KT Jeang Retrovirology Prize.

**Table:** KT Jeang Retrovirology Prize: the new committee

Name	Member of Retrovirology Editorial Board	Location
Karen Beamon	No	USA
Monsef Benkirane	Yes	France
Ben Berkhout	Yes	Netherlands
Seph Borrow	Yes	UK
Carol Carter	No	USA
Michael Emerman	No	USA
Ari Fassati (chair)	Yes	UK
Masahiro Fujii	Yes	Japan
Thumbi Ngund’u	Yes	South Africa
Monique Nijhuis	Yes	Netherlands
Leslie Parent	Yes	USA
Susan Ross	Yes	USA

